# The Role of Neutrophils in Spondyloarthritis: A Journey across the Spectrum of Disease Manifestations

**DOI:** 10.3390/ijms24044108

**Published:** 2023-02-18

**Authors:** Lavinia Agra Coletto, Chiara Rizzo, Giuliana Guggino, Roberto Caporali, Stefano Alivernini, Maria Antonietta D’Agostino

**Affiliations:** 1 Division of Rheumatology, Università Cattolica del Sacro Cuore, Policlinico Universitario Agostino Gemelli IRCSS, 00168 Rome, Italy; 2 Department of Health Promotion, Mother and Child Care, Internal Medicine and Medical Specialties, Rheumatology Section, University of Palermo, 90127 Palermo, Italy; 3 Division of Clinical Rheumatology, ASST Gaetano Pini-CTO Institute, 20122 Milan, Italy; 4 Department of Clinical Sciences and Community Health, University of Milan, 20122 Milan, Italy

**Keywords:** spondyloarthritis, neutrophils, psoriasis, uveitis, enthesitis, arthritis, inflammatory bowel disease

## Abstract

Spondyloarthritis (SpA) contemplates the inflammatory involvement of the musculoskeletal system, gut, skin, and eyes, delineating heterogeneous diseases with a common pathogenetic background. In the framework of innate and adaptive immune disruption in SpA, neutrophils are arising, across different clinical domains, as pivotal cells crucial in orchestrating the pro-inflammatory response, both at systemic and tissue levels. It has been suggested they act as key players along multiple stages of disease trajectory fueling type 3 immunity, with a significant impact in the initiation and amplification of inflammation as well as in structural damage occurrence, typical of long-standing disease. The aim of our review is to focus on neutrophils’ role within the spectrum of SpA, dissecting their functions and abnormalities in each of the relevant disease domains to understand their rising appeal as potential biomarkers and therapeutic targets.

## 1. Introduction

The term Spondyloarthritis (SpA) includes a group of different diseases sharing a common pathogenetic background and similar clinical features such as Ankylosing Spondylitis (AS), Psoriatic Arthritis (PsA), inflammatory-bowel-disease-related arthritis (IBD-related arthritis), and Reactive Arthritis (ReA) [1].

**Figure 1:**
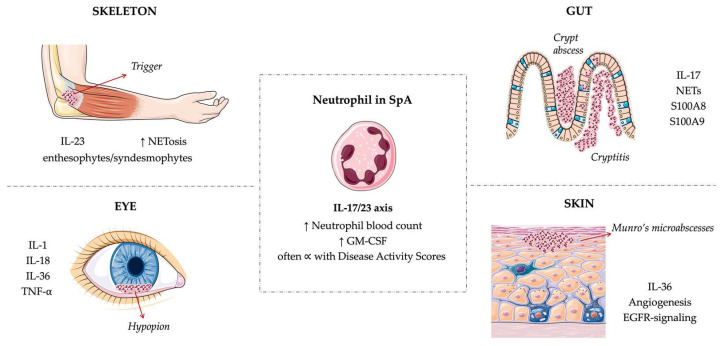
Legend. Neutrophils are pleiotropic cells that play a central role in the very early phase of SpA development, contributing to triggering inflammation in multiple tissues. Neutrophils are recruited from the bloodstream to target sites in Spondyloarthritis (SpA), such as joints, entheses, gut, skin, and eyes, where they produce neutrophils extracellular traps (NETs), cytokines, and chemokines to attract and activate other immune cells, boosting type 3 immunity.

Skeletal manifestations involve both spine and peripheral joints, with a prominent role played by enthesis within the so-called synovio–entheseal complex, which is the anatomical structure linking tendons or ligaments to bones [2]. Extra-articular features of SpA are mostly represented by skin disease, with psoriasis or psoriasiform lesions, gut inflammation, both clinical and subclinical, and eye involvement, characteristically evolving in uveitis [3]. From a pathogenetic point of view, aberrant innate and adaptive immune responses, triggered by several environmental factors in a genetically predisposed subject, determine the development of a strong pro-inflammatory milieu that drives the systemic inflammation retrieved in SpA [4]. Given the absence of detectable serum autoantibodies and the relevance of Human Leucocyte Antigen (HLA) and T cells in the pathogenesis, SpA, as a matter of classification, has touched in the past the fine line of the autoinflammatory diseases spectrum, where innate immunity leads the way [5]. In the context of recent advances, amidst the three immunity types which reshaped the categorization of immune response [6], disrupted type 3 immunity stood out as the most pervasive trait in SpA pathophysiology [7]. Physiologically, extracellular disturbances in barrier tissues, for instance, caused by the interaction with bacteria and fungi, initiate type 3 response, where interleukin (IL)-17 and IL-22 orchestrate cellular compartments to protect the body against the damage with the aim of restoring tissue homoeostasis [6]. However, when abnormal, they contribute to SpA pathogenesis by amplifying T helper 17 cells (Th17) response, hence recruiting and activating neutrophils at different disease sites (enthesis, joint, gut, eye, and skin) in a positive loop, further amplified by neutrophilic production of pro-inflammatory mediators, such as IL-23 [8]. Hereby neutrophils are emerging as pivotal cells in SpA development, being involved in several manifestations, especially in the very early phase of the disease (Figure 1).

In the figure, the typical accumulation of neutrophils at the tissue level in the form of a cryptic abscess, Munro’s microabscess, and hypopyon is depicted within the intestinal mucosa, the skin, and the eye, respectively. At the entheseal level, the influx of neutrophils and their consequent activation drive enthesitis development and cooperate with the occurrence of new bone formation. Interleukin (IL), Tumor Necrosis Factor (TNF), Granulocyte-Macrophage Colony Stimulating Factor (GM-CSF).

### Neutrophils Overview

Every day, 10^11^ neutrophils are produced by the human bone marrow, making them the most abundant circulating leucocyte population, known as an essential component of innate immunity [9]. Due to their distinctive short lifespan, they have been historically perceived as a homogeneous and transcriptionally inactive cell population made for quickly responding to microbial and sterile challenges covering defensive and inflammatory duties. In the bone marrow, the maturation process from granulocyte–monocyte progenitors to segmented neutrophils is conducted by the granulocyte colony-stimulating factor (G-CSF). Once mature, neutrophils are mobilized from the bone marrow reservoir, upregulating CXC chemokine receptor 2 (CXCR2) and Toll-like receptor 4, whose ligands are expressed by endothelial cells [10], and downregulating the integrin α4β1 (VLA4) and the CXCR4 which usually binds resident stromal cells via vascular cell adhesion molecule 1 (VCAM1) and chemokine stromal-derived factor-1/SDF-1 (CXCL12), respectively [11].

Outside the bone marrow, the network orchestrating neutrophil function expands, and among the key regulators, IL-23 and IL-17 play a significant role in their activation [12]. Neutrophils themselves are capable of producing IL-17, perpetuating positive feedback with Th17 cells [13].

Hence, the interaction with activated endothelium via selectin and adhesion molecules together with a gradient of chemoattractant mediates neutrophils migration, adhesion, and diapedesis into inflamed tissue where they exert their function of clearance of extracellular pathogens or cell debris by multiple mechanisms, including phagocytosis and the release of reactive oxygen species (ROS), granules, and neutrophils extracellular traps (NETs).

Such static archetype has been challenged when neutrophils proved to modulate at different levels of innate and adaptive immunity, hence not limiting their role in the initiation and amplification of the inflammatory response [14]. Indeed, neutrophils may behave as antigen-presenting cells, able to produce chemokines, cytokines, exosomes, and granules targeting endothelium, dendritic cells, complement, macrophages, and lymphocytes with the result of upregulating or downregulating their function [15]. They were shown to populate secondary lymphoid organs under both homeostatic and inflammatory conditions, colonize peri-marginal zone areas of the spleen, display a B-cell-helper function contributing to immunoglobulin diversification, and engage bi-directional interaction with various subsets of T cells [15,16].

The advent of new techniques, such as single-cell RNA-sequencing (scRNA-seq) and pseudo-time analysis, revealed that functional diversity was mirrored by phenotypical diversity, starting to shape the heterogeneous trajectory of neutrophilic phenotypes, capable of aging and plasticity, both in health and disease [17,18].

Deregulation of neutrophils causes damage, both when defective, by worsening sepsis consequences, or hyperactive, contributing to tissue damage in diverse conditions, comprising cancer and immuno-mediated diseases such as SpA [18]. A comprehensive knowledge of neutrophil biology, functions, and deregulation in SpA may contribute to a deeper understanding of disease onset with a consequent important impact on the discovery of new therapeutic targets in SpA. In this narrative review, considering the pleiotropic role of neutrophils, we aim to evaluate their behavior in SpA, dissecting their function and abnormalities in each of the relevant disease domains.

## 2. Neutrophils across SpA Manifestations

### 2.1. Articular Involvement

#### 2.1.1. Enthesis

SpA clinical picture is dominated by significant articular and periarticular involvement, and enthesitis is considered the hallmark feature. Specifically, enthesis seems to be the primary site of inflammation responsible for the systemic activation of the immune system, and neutrophils are deeply involved in the very early phase of disease, as they mediate tissue damage through ROS and protease production [19].

Resident cells release pro-inflammatory mediators, including G-CSF, IL-17, IL-8, and prostaglandins, to induce vasodilation of the transcortical feeding vessels and to favor the active recruitment and diapedesis of neutrophils in loco [20,21]. This specific crosstalk is exemplified by the evidence of stromal cells, obtained from enthesis specimens, that once stimulated with fungal adjuvants, release IL-8 and upregulate leukocytes adhesion molecules, such as VCAM-1 and Intercellular Adhesion Molecule-1 (ICAM-1), key contributors to neutrophils migration.

Notably, Tamassia N. and colleagues showed, for the first time, the presence of neutrophils in healthy spinal peri-entheseal bone. These enthesis-derived neutrophils stimulated in vitro were able to release a significant amount of IL-23, a key cytokine in SpA pathogenesis and enthesitis development [22].

The presence and activation of neutrophils, together with a marked increase in the alarmins S100A8 and S100A9, were elegantly showed by Stavre Z. in *SKG mice* withcurdlan-indiced enthesitis, a common use animal model for chronic arthritis [23,24]. These aforementioned alarmins constitute calprotectin, a biomarker of inflammation for both SpA and IBD, in close relationship with the development of tendinopathy, SpA structural damage, psoriasis, and gut inflammation [25,26,27]. S100A8 and A9 may also have an autocrine effect, stimulating their margination and adhesion to the vessel wall to translocate into inflamed tissue. Thus, alarmin production could sustain neutrophil influx to enthesis in the early phase of inflammation [28].

In addition, the proinflammatory cytokine burst, evidenced in myeloid cells during enthesitis, seems to be related to hyperreactive signaling via Signal Transducer and Activator of Transcription 1 (STAT1), and in the absence of the STAT1 counterregulatory protein A20, entheseal inflammation occurs spontaneously [29], strengthening the relevance of myeloid compartment in enthesitis pathophysiology.

Obtaining enthesis specimens to perform histological and functional assays is challenging [30]; however, a new minimally invasive technique to perform entheseal biopsy of the elbow common extensor tendon was recently described. The authors not only showed the ability to sample proper entheseal tissue but also demonstrated the presence of CD45^+^ cells in the digested tissue [31]. This finding paves the way to the possibility of reaching a comprehensive characterization of the entheseal cellular environment.

#### 2.1.2. Peripheral and Axial Joints

Together with enthesitis, SpA patients classically suffer from joint inflammation at both axial and peripheral levels. The prevalent involvement of the spine and sacroiliac joints defines the axial SpA (axSpA) phenotype; while in the presence of mainly oligoarthritis or polyarthritis and in the absence of axial involvement, the disease is classified as peripheral SpA (pSpA) [32].

Histological data reported the presence of neutrophils at both levels, pointing out a potential role in triggering the initial inflammatory cascade in SpA. In specimens from early sacroiliitis, neutrophils were evidenced among other immune cells, taking part in the enthesitis process in such areas [33]. In addition, in facet joints tissue of AS patients, a strikingly high number of IL-17^+^ neutrophils, as demonstrated by double immunofluorescence assays with both CD15 and Myeloperoxidase (MPO), was found, suggesting a role even in the advanced stage of disease and particularly in the occurrence of syndesmophytes and enthesophytes [34].

Sampling tissues from the axial skeleton is difficult, but neutrophils presence and activity in SpA spinal inflammation and consequent structural changes are inferable by the increased levels of Granulocyte-Macrophage Colony Stimulating Factor (GM-CSF) in patients with persistently active disease [35]. Indeed, enhanced granulopoiesis and neutrophil differentiation are evidenced in inflamed tissues of SpA patients, and they are mirrored peripherally in the blood by the increase in neutrophil count, findings confirmed in animal models of disease [36]. Neutrophil-to-leukocyte ratio, when elevated, has been proposed as a marker of disease activity, linking neutrophils to clinical manifestations of SpA and disease activity scores [37] and the increased number of granulocytes precursors, as a marker for structural damage in axSpA [38].

From a functional point of view, in the peripheral blood of patients affected by radiographic axSpA with active clinical disease, a spontaneous excessive Neutrophil Extracellular Traps (NETs) generation was depicted with an increase in NETs remnants, such as cell-free DNA, cell-free nucleosomes and elastase [35]. These NETotic products correlate with inflammatory markers and may be used to discriminate active patients [39]. Notably, NETs obtained from AS patients are enriched with bioactive IL-17A and IL-1β. IL-17A induces the differentiation of mesenchymal stem cells toward bone-producing cells, whereas IL-1β contributes, in a pro-inflammatory loop, to determine IL-17 accumulation in NETs. To reinforce this concept, the blockade of the IL-1 β pathway reduces the osteogenetic differentiation of mesenchymal stem cells [40].

Taken together, these lines of evidence may pinpoint an active role for neutrophils and their upstream stimulation in SpA initiation and progression, implying an unrecognized plasticity of these cells across SpA phases with specific involvement in spinal damage [41,42].

Peripheral SpA may account for a neutrophil role as well. The first description of neutrophils in synovial fluid from AS-inflamed knees dates back to 1973 [43]. Later, tissue analysis of synovial membrane from inflamed joints revealed the presence of neutrophil infiltrate. Polymorphonuclear leukocytes (PMNs) infiltrates are comparable among all SpA subsets (axial and peripheral), including PsA, and are increased when compared to rheumatoid arthritis [44].

In active SpA, PMNs infiltration showed a strong correlation with C-Reactive Protein (CRP) and Erythrocyte Sedimentation Rate (ESR), demonstrating a close relationship with systemic inflammation and clarifying that local inflammation mirrors global disease activity [45]. Moreover, the presence of abundant PMNs infiltrate in joints of all SpA subtypes in the initial phase of disease reinforces the concept that innate immunity may act as the primary driver in determining peripheral joint inflammation. On the other hand, the reduction of PMNs at the joint level is obtained after treatment, as demonstrated with TNF inhibitors in early SpA [46], making histological changes a useful biomarker to assess response to treatment.

Recently, a marker of NETosis, the MPO–DNA complex, was shown to be increased in PsA peripheral blood as compared to PsO, suggesting a stronger inflammatory burden in PsA and a more robust occurrence of NETosis in such patients. The MPO–DNA complex correlated with disease activity parameters in PsA, especially with tender and swollen joint counts, as well as with PsA disease activity scores (DAPSA, Disease Activity in PSoriatic arthritis), indicating that its serum fluctuation may serve as a marker to catch articular involvement, and even response to drugs [47]. NETs in the context of PsO were demonstrated to induce Th17 cells, with a stronger induction in the presence of TRAF3 Interacting Protein 2 (TRAF3IP2) genotype [48], renowned for increasing the susceptibility of developing PsA and associated with a more severe joint involvement in PsA [49]. We can hypothesize that, as observed in PsO, NETs may enhance Th17 response in PsA, contributing to orchestrating tissue and systemic inflammation via the IL-17 axis. Indeed Cathelicidin Antimicrobial Peptide LL 37 (LL37), a product of neutrophil degranulation, is highly represented in PsA synovium [50]. Neutrophil degranulation is boosted in the presence of the complement factor, C5a, and of GM-CFS, both increased in PsA synovial tissue. Once released, LL37 may elicit the generation of anti-LL37 autoantibodies that can contribute to shape the pro-inflammatory milieu in PsA favoring immune-complex (IC) deposition, that in turn fuels NETosis in a self-maintained loop. Indeed, neutrophils were shown to be IC^+^ synovial cells, presenting co-localization of IgG and LL37 on immunofluorescence examination, mainly in synovial tissue of PsA patients hosting lymphoid aggregates, so they could act as source of antigens and, simultaneously, as target of antibody-mediated immune response [51].

### 2.2. Gut Involvement

The link between gut inflammation and SpA emerged in the last decades following the clinical observation that up to 10% of SpA patients develop IBD, mainly as Crohn’s disease (CD), and more than 50% present subclinical gut inflammation at histological examination, without overt IBD manifestations [52]. The actual knowledge of neutrophil functions in gut inflammation is still incomplete. Evidence from IBD studies pointed out a double-edged behavior of these cells, as they can contribute to inflammatory changes and tissue damage and, simultaneously, to tissue healing and inflammation resolution [53]. In CD, crosstalk between Th17 and neutrophils was recently described, where the two populations of cells co-localized in the mucosa, suggesting a vicious bidirectional loop [13]. A similar mechanism, if present in SpA, could contribute to the IL-17-driven inflammation evidenced in AS and PsA.

The implication of neutrophils in driving gut inflammatory changes in SpA patients may be inferred by the close relationship with microbiota dysbiosis and the presence of an altered gut-vascular barrier (GVB), making easier the translocation of pathogens and their products to the lamina propria with the consequent triggering of immune responses [54,55]. Specifically, NETs were shown to further elicit bacterial translocation from the lumen and promote the apoptosis of epithelial gut cells, contributing to the disruption of intestinal barrier integrity [56].

The integrity of GVB relies on type 3 immunity with a delicate equilibrium of cytokine production to grant tight junction functions, mucus production, and, ultimately, gut homeostasis [57,58]. SpA patients exhibit a well-known unbalance in type 3 cytokine response, specifically IL-23, IL-17, and IL-22, which contributes to the disruption of gut epithelial integrity resulting in the occurrence of the so-called “leaky gut” [59]. Neutrophils are attracted by IL-8 and actively released by resident epithelial cells.

Once there, they can be considered both initiators of the inflammatory process and active contributors of mucosal damage by recognizing Pathogen Associated Molecular Patterns (PAMPs), acting as antigen-presenting cells (APCs), releasing toxic molecules (including reactive oxygen species (ROS), matrix metalloproteases (MMPs), and NETs), cytokines (such as CXCL8 and IL-17) and alarmins, ultimately boosting a strong pro-inflammatory cascade and shaping the inflammatory microenvironment [60,61,62]. The presence of neutrophils in the inflamed intestinal mucosa is corroborated by increased levels of calprotectin (S100A8 and S100A9) in SpA feces and serum [63] and by histopathological findings. Indeed, in SpA subclinical ileal inflammation, an acute form of colitis strikingly resembling bacterial enterocolitis was found, with massive influx of PMNs within the lamina propria and the epithelium [64]. Such picture is retrieved in both AS and PsA [65] and correlates with a predominant peripheral disease. On the other hand, chronic changes, accounting for a profound architectural distortion of the mucosal ultrastructure in the presence of immune infiltrate and aggregates in the lamina propria, are characteristically related to established axial disease [66,67].

However, neutrophils exert scavenger functions and may directly produce anti-inflammatory cytokines, such as IL-10 and resolvins, that mediate tissue remodeling and mucosal healing [68,69]. The counter-regulation of their activity is mainly determined through apoptosis and consequent reduction of neutrophil infiltrate [70,71].

Notably, the presence of an increased level of GM-CSF in IBD favors neutrophil survival, inhibiting apoptosis [72,73]. GM-CSF is upregulated even in SpA, and a common detrimental function on neutrophil homeostasis may then be present in this condition as well.

Neutrophils display tissue-specific and microenvironment-driven functions; they are short-lived cells recruited within minutes with a peak response occurring in 24–48 h, thus hard to study in action [74]. However, their effects go beyond such a time frame since they favor the local polarization of immune cells, which can recirculate from the intestinal niche to target sites of disease, including joints, enthesis, eyes, and skin [75].

### 2.3. Psoriasis

PsO is an inflammatory skin disease rather prevalent (1–5%) worldwide [76] and in approximately 10 to 30% is associated with or may precede PsA occurrence [77]. The crosstalk between innate and adaptive immune systems leads to a self-perpetuating inflammatory loop played by T cells (mainly Th1, Th17, and Th23 driven), dendritic cells, keratinocytes, and neutrophils [78].

In the skin, neutrophils are the earliest cells present in psoriatic plaques, and when clustering, they are responsible for pathognomonic histological hallmarks, such as Munro’s microabscesses and Kogoj spongiform micropustules [76].

In the blood of psoriatic patients, neutrophils are augmented in terms of absolute count [79], ratio (neutrophil-to-lymphocyte ratio) [80], and of subsets (low-density granulocytes (LDG) and normal-density granulocytes (NDG). Both the absolute count and circulating LDG are associated with psoriasis skin disease severity (PASI: Psoriasis Area Severity Index) [81]. Cytokines associated with neutrophil recruitment, differentiation, activation, and release are increased in the plasma of psoriasis patients compared to controls [79].

Conversely, therapeutic depletion of myeloid lineage leukocytes, whether voluntary or involuntary, is associated with a rapid improvement of psoriasis [82,83], and targeted biologic therapies for psoriasis simultaneously ameliorate clinical symptoms and normalized neutrophil activity and count [84].

Neutrophil alterations are observed at all stages, comprising respiratory burst, degranulation process, and NETosis [85].

They produce and release a larger amount of ROS compared to healthy individuals. The activity of NADPH oxidase (NOX2) and MPO, two key enzymes responsible for the respiratory burst, is increased both in the skin and serum of PsO patients and correlates with PASI severity [86,87]. Enzymes stored and then released by neutrophils granules are implicated into the pathogenesis of psoriasis: (i) proteinase 3 is involved in the proteolytical activation of inflammatory mediators (such as IL-36 and Tumor Necrosis Factor (TNFα)) [78,88] and in the formation of autoantigens in psoriasis (LL37) [89], contributing both to local and systemic inflammation; (ii) neutrophil elastase (NE) enhances keratinocyte proliferation via proteolytic activation of epidermal growth factor receptor (EGFR) signaling [90] and together with (iii) cathepsin G contribute to IL-36 activation and subsequent skin inflammation [88]; (iv) the relevance of MPO, already mentioned for its role in the respiratory burst, is also evinced by genetic and functional studies where the gene encoding for MPO resulted as a genetic determinant of generalized pustular psoriasis and neutrophil abundance [91]; (v) finally lipocalin 2 (LCN2) is an antimicrobial protein which seems to enhance Th17-mediated inflammation, and whose serum level is elevated in psoriatic patients and correlate with the severity of itching [92].

Finally, NETosis is more than a bystander effect of neutrophil dysregulation in psoriasis. NETs are increased both in psoriatic lesions [93,94] and blood samples of psoriatic patients, where they correlate with PASI [95,96]. They act as key players in inducing and maintaining inflammation in different manners: (i) DNA-LL37 complexes stimulate the production of Interferon-alpha (IFN-α) and IFN-β by plasmacytoid dendritic cells (pDCs) [97], whereas (ii) RNA-LL37 complexes stimulate TNF-α and IL-6 production by myeloid DCs [98,99], (iii) NETs signaling, through activation of Toll-Like Receptor-4 (TLR-4) and IL-36, amplifies skin inflammation [94] and (iv) IL-17A, a central cytokine in psoriasis pathogenesis, is released by neutrophils during the formation of NETs in psoriatic lesions [100].

To close the loop, gene ontology enrichment analysis of genes expressed in psoriatic lesions also revealed significantly upregulated genes involved in neutrophil modulation [101], reinforcing neutrophils’ relevance in the pathogenesis of psoriasis.

### 2.4. Uveitis

Uveitis is the most common extra-articular manifestation in SpA, occurring preferentially in *HLA-B27*-positive patients. Up to 50% of AS and only 7% of PsA, and 2–5% of IBD-SpA patients develop uveitis [102].

Uveitis can be classified as anterior, intermediate, or posterior, according to the portion of the eye involved in the inflammatory process. In the case of global inflammation, the disease is described as panuveitis. The direct detection of leukocytes in a fluid near the uveal tract or the visualization of choroid or retina lesions allows the diagnosis [103]. Uveitis displays differential clinical presentations across SpA: in AS, it is classically anterior, unilateral, recurrent, more frequent in males and with sudden onset; while in PsA, it is anterior and intermediate, bilateral, chronic, affecting more often females and with an insidious onset [104].

As evidenced in studies of patients with acute anterior uveitis (AAU) and animal models, innate immunity cells, including neutrophils, play a role in both SpA and ocular autoimmune disease [105].

In the early phase of the disease, neutrophils and macrophages massively infiltrate ocular structures with concurrent edema, increase in vascular permeability, and congestion. In acute uveitis, neutrophils secrete several proinflammatory cytokines, such as IL-1, IL-18, IL-36, and TNFα, as well as ROS and lysosomal enzymes [106]. Especially in *HLA-B27* subjects, they contribute to the accumulation of such proinflammatory cytokines in the aqueous humor taking part in non-granulomatous inflammation [107] and rarely, in the case of tissue necrosis, giving rise to real abscesses.

In acute uveitis and in case of relapsing disease, hyperreactive neutrophils may be responsible for tissue damage [108] and for triggering an inflammatory loop that can simultaneously affect the eye, joints, and skin. In detail, the pathophysiology of anterior uveitis generally involves the presence of chemoattractants in the corneum and the hyperreactivity of neutrophils via the increase in superoxide production and chemotaxis, as evidenced in models of AAU [109,110]. A nice report on a PsA patient demonstrated the link between peripheral neutrophil activation and eye disease [111]. In particular, iridocyclitis characterized by severe hypopyon (reflecting neutrophil infiltration of the anterior chamber) was coupled with the occurrence of arthritis and the worsening of psoriatic lesions. Moreover, the peripheral neutrophils of the patient were shown to be in an activated state during the disease flare and normalized after treatment.

An aberrant response to bacterial molecules in neutrophils has been proposed as a leading mechanism in the pathogenesis of AAU. In active AAU, including AAU related to SpA, there is a selective perturbation in the expression and function of TLR-2 and TLR-4. Such receptors are involved in recognition of bacterial PAMPs, namely lipoproteins and LPS, and can be internalized after the engagement with their ligands. In peripheral neutrophils obtained during AAU flare, a significant reduction in the levels of TLR-2 expression was observed, suggesting that neutrophils may have been activated by microbial products with consequent downregulation of surface receptors. Moreover, in vitro stimulation of patients’ neutrophils with TLR ligands resulted in an increased production of IL-1, especially after TLR-2 activation [112]. The observation that LPS derived from Chlamydia trachomatis (a pathogen strongly implicated in *HLA-B27* associated AAU and ReA) signals via TLR-2 strengthens the hypothesis of a possible alteration in neutrophils response to PAMPs and the occurrence of uveitis in the context of SpA [113,114].

The role of neutrophils in ocular inflammation is further evinced from studies on experimental autoimmune uveoretinitis. In particular, G-CSF and G-CSF-R are elevated in the ocular tissue and blood of patients with uveitis [115]. G-CSF may act both at the bone marrow level, determining an increased mobilization of neutrophils with consequent neutrophilia, and at the tissue level, enhancing sterile inflammation mediated by neutrophils. In this regard, ocular endothelial cells and Th-cells are accounted as a source of G-CSF, which leads to the release of neutrophils chemoattractants, such as CXCL2 and IL-8 [10]. Local G-CSF favors the expression of adhesion molecules in the endothelium itself and of chemokine receptors on neutrophils (CXCR2 and CXCR4), thus promoting their trafficking to the inflamed eye and, at the same time, boosting their survival [116]. Moreover, G-CSF-activated-neutrophils discharge proinflammatory cytokines, including IL-17 and IL-1, that influence Th17 polarization via effects on dendritic cells and IL-23 release [100,117], highlighting the inflammatory circle described in the pathogenetic process typical of SpA.

Intriguingly, the genetic deletion of G-CSF or the administration of monoclonal antibodies selectively blocking G-CSF caused a profound reduction in the severity of ocular manifestation in uveoretinitis models [118]. The decreased activation of neutrophils impaired even the differentiation of pathogenic Th17, bridging the two branches of immunity and showing up the fascinating hypothesis that neutrophils are more than ancillary cells during sterile inflammation, being able to actively contribute to uveitis development.

To conclude, a clue for neutrophil involvement in uveitis related to SpA can be inferred from anatomical studies, as McGonagle and colleagues proposed that the connective tissue of uveal structures, especially at the level of ciliary body tendons attachment, may be considered as a classical musculoskeletal enthesis. The same group, as mentioned above, recently described the presence of neutrophils at entheses so that we can speculate a possible common immunologic milieu for skeleton and eye structures, both contributing to SpA manifestations [24].

Taken together, these findings suggest neutrophils as key players in the systemic inflammatory process of SpA, intertwining different niches [111,119].

How neutrophils can be triggered in SpA to determine marked inflammation across multiple tissues is a matter of debate. In this regard, an intriguing unifying theory has emerged in the last few years accounting for a link between gut and eye inflammation, the so-called gut–eye axis [120]. This observation in SpA is coupled with the renowned hypothesis of the gut–joint axis in which the leaky gut may account for the spreading of bacterial antigens to several tissues, including the intraocular environment, as well as for the local activation of immune cells that can then recirculate to target sites of disease [121].

## 3. Therapeutics: Neutrophil-Targeted Therapies

Given the cross relevance of neutrophils among the most diverse pathologies with a huge epidemiological impact (such as sepsis, oncologic and autoimmune diseases), pharmacological research has implemented the development of drugs aimed at extinguishing, enhancing or modulating neutrophils count, function, or phenotype, when derailed [122].

We addressed the pathogenetic abnormalities concerning neutrophils across the various domain in the spectrum of SpA, and the main strategy to counterbalance the damage caused by neutrophils would be to switch off or attenuate their functionality, trying to contextualize them within the delicate cellular crosstalk.

This could be achieved by several approaches, including directly modulating neutrophils production, maturation, release into the circulation, migration, and accumulation in target inflamed tissues; by regulating their vitality or their mediators; and by indirectly modulating transduction signaling, albeit not specific only for neutrophils.

These are summarized in Table 1, with a special focus on drugs approved or attempted in clinical trials conducted on patients affected by autoimmune diseases.

Results are overall promising; however, some challenges, especially concerning safety and selectivity, remain. The major concern about safety is related to the risk of exacerbating severe infections, which is a recurrent challenge for rheumatologists when administering immunosuppressive therapies. Nonetheless, drug-induced neutropenia not always means interference with neutrophil-mediated antimicrobial host defense, as reported, for example, for IL-6 inhibitors and Janus Kinase (JAK) inhibitors [123,124], where neutropenia could serve as an indicator of efficacy without necessarily mirroring an increased rate of infections [125].

When targeting neutrophils, selectivity, and specificity represent the major technical challenge, which needs to be better addressed. Given the similarities and overlapping regulation of neutrophils with the other myeloid lineages, including macrophages, dendritic cells, monocytes, and osteoclasts, side effects might include disturbance of immunity and possibly bone metabolism.

Increasing the knowledge and characterization of neutrophil subsets and phenotypes [126] will help target specific neutrophil subpopulation, hopefully narrowing the desired effect on the pathological phenotype or dysfunction.

**Table 1:** Inhibition of neutrophils: therapeutic approaches in autoimmune diseases.

Strategies	Approaches	Target	Drugs and Diseases
Reducing neutrophil numbers	Targeting production	- GM-CSF receptor	*Mavrilimumab* in GCA and RA [127,128,129]
		- GM-CSF	*Otilimab* in RA [130]; *Namilumab* in RA [131], PsO [132] and SpA (NCT03622658); *Gimsilumab* in AS (NCT04205851; NCT04351243)
		- IL-23/IL-17 axis (a regulator of G-CSF production)	*IL-17 inhibitors* in PsO, PsA, ax-SpA; *IL-12/23 inhibitors* in PsO, PsA [133], CD, and SLE; *IL-23 inhibitors* in CD, UC, PsO, and PsA
	Inducing depletion	- Circulating neutrophils	-*Extracorporeal granulocytapheresis* in RA [134], CD, and RCU [135]
Interfering with neutrophil recruitment and chemotaxis	Selectin and integrin blockers	- α4β1-integrin	*-Natalizumab* in CD [136]
		- Selectins	-TBC1269 (and others) in PsO [137]
	Blocking complement	- C5a and C5a receptor	*-Eculizumab* and *Avacopan* in AAV [138,139]; *NNC0215-0384* in RA (NCT01611688)
	Blocking leucotriens	- LTB4	-*CP-195543* in RA (NCT00424294)
Blocking neutrophils activation	Signal transduction blockade (cytokine signaling in neutrophils)	- JAK	-*Jak-inhibitors* in PsA, RA, UC, AS [140]
		- SYK	-*Fostamatinib* in RA [141] and SLE [142]
		- PDE4	-*Apremilast* in PsO, PsA [143], SLE (NCT00708916) and AS [144]
	Blocking cytokines whose receptors are also on neutrophils	- TNF-α	-*TNF-α inhibitors* in RA, PsA, PsO, SpA, AS, CD, UC, uveitis [145]
		- IL-6	-*IL-6 inhibitors* in RA, AS, SSc, vasculitis, SLE, AOSD [146]
Blocking neutrophil-derived mediators	Neutrophil granule enzymes	- MMP9	-*andecaliximab* in UC and CD [147,148]
	NETs (blocking activity of enzymes critical for NETs formation)	- NADPH, MPO, PAD4, DNase Inhibitor	Not yet elucidated in humans affected by autoimmune diseases.
Others	Blocking neutrophil function	- Neutrophil inflammasome	-*β-hydroxybutyrate* in gout flares [149] -*IL-1β inhibitors* in RA, SpA, PsA, AS, AOSD, uveitis, GCA, vasculitis [150]
		- Neutrophils alarmins (S100A8/S100A9)	-*Paquinimod* in SLE (NCT00997100)

Abbreviations: Granulocyte-Macrophage Colony Stimulating Factor (GM-CSF), Interleukin (IL), (LTB4), (JAK), (SYK), Tumor Necrosis Factor (TNF), phosphodiesterase-4 (PDE4), Metalloproteinase (MMPS), Nicotinamide adenine dinucleotide phosphate (NADPH), ANCA-Associated Vasculitis (AAV), Myeloperoxidase (MPO), Rheumatoid Arthritis (RA), Psoriatic Arthritis (PsA), Psoriasis (PsO), Spondyloarthritis (SpA), Ankylosing Spondylitis (AS), Ulcerative Colitis (UC), Crohn Disease (CD), Giant Cell Arteritis (GCA), Adult Onset Still’s Disease (AOSD), Systemic Lupus Erythematosus (SLE).

## 4. Conclusions

The definition of SpA itself is a tough task due to the extreme heterogeneity of clinical manifestations, where apparently distant organ involvements are each defined by one specific disease. Yet, joints, spine, gut, skin, and eyes diseases are often clinically associated, embracing one another and sharing a common pathogenic background, driven by type 3 immunity, as recently demonstrated by Nakamura et al. [151].

In this complex and delicate picture, neutrophils designate a common thread, being a cell cross-sectionally involved in the pathogenesis within each of the addressed domains.

They indeed represented the bulky inevitable cell, depicting pathognomonic lesions, faithfully responding to the acute inflammatory duties. Their peripheral count and biological functions were shown to be affected by multilayer evidence both at early and late stages of the disease, often mirroring disease activity.

Moreover, their pathogenic relevance increased, considering their multifaced role, not only as soldiers of the innate immune system but also capable of regulating homeostatic functions and engaging a bidirectional dialogue with adaptive immunity [152]. Neutrophils were shown to be capable of releasing a larger amount of IL-17A and of acting as APCs for T cells into already inflamed tissues [153,154]. Taken together, such evidence, coupled with the reviewed data, stress neutrophils once again as pivotal in SpA pathogenesis, making them attractive therapeutic targets, with the opportunity of exploiting diverse strategies and approaching all stages of their regulation.

Nonetheless, emergent technologies, both in the outpatient setting and the wet lab, are paving the way for deepening the knowledge on neutrophils’ pathophysiology and across SpA disease course, getting closer to the definition of novel biomarkers and treatment options.

## Data Availability

Not applicable.

## Abbreviations

AS, Ankylosing Spondylitis; axSpA, axial SpA; CD, Crohn’s Disease; G-CSF, Granulocyte-Colony Stimulating Factor; GM-CSF, Granulocyte-Macrophage Colony Stimulating Factor; IBD, Inflammatory Bowel Disease; IL, Interleukin; NETs, Neutrophils Extracellular Traps; pSpA, peripheral SpA; PsA, Psoriatic Arthritis; PsO, Psoriasis; ReA, Reactive Arthritis; ROS, Reactive Oxygen Species; SpA, Spondyloarthritis; UC, Ulcerative Colitis.

## References

[1] Dougados M., Baeten D. (2011). Spondyloarthritis. Lancet.

[2] Benjamin M., McGonagle D. (2001). The anatomical basis for disease localisation in seronegative spondyloarthropathy at entheses and related sites. J. Anat..

[3] Bengtsson K., Forsblad-D’Elia H., Deminger A., Klingberg E., Dehlin M., Exarchou S., Lindström U., Askling J., Jacobsson L.T.H. (2021). Incidence of extra-articular manifestations in ankylosing spondylitis, psoriatic arthritis and undifferentiated spondyloarthritis: Results from a national register-based cohort study. Rheumatology.

[4] Ronneberger M., Schett G. (2011). Pathophysiology of Spondyloarthritis. Curr. Rheumatol. Rep..

[5] Generali E., Bose T., Selmi C., Voncken J.W., Damoiseaux J.G. (2018). Nature versus nurture in the spectrum of rheumatic diseases: Classification of spondyloarthritis as autoimmune or autoinflammatory. Autoimmun. Rev..

[6] Annunziato F., Romagnani C., Romagnani S. (2015). The 3 major types of innate and adaptive cell-mediated effector immunity. J. Allergy Clin. Immunol..

[7] Mauro D., Simone D., Bucci L., Ciccia F. (2021). Novel immune cell phenotypes in spondyloarthritis pathogenesis. Semin. Immunopathol..

[8] Macleod T., Bridgewood C., McGonagle D. (2023). Role of neutrophil interleukin-23 in spondyloarthropathy spectrum disorders. Lancet Rheumatol..

[9] Ng L.G., Ostuni R., Hidalgo A. (2019). Heterogeneity of neutrophils. Nat. Rev. Immunol..

[10] Eash K.J., Greenbaum A., Gopalan P.K., Link D.C. (2010). CXCR2 and CXCR4 antagonistically regulate neutrophil trafficking from murine bone marrow. J. Clin. Investig..

[11] Summers C., Rankin S.M., Condliffe A.M., Singh N., Peters A.M., Chilvers E.R. (2010). Neutrophil kinetics in health and disease. Trends Immunol..

[12] Rosales C. (2018). Neutrophil: A Cell with Many Roles in Inflammation or Several Cell Types?. Front. Physiol..

[13] Pelletier M., Maggi L., Micheletti A., Lazzeri E., Tamassia N., Costantini C., Cosmi L., Lunardi C., Annunziato F., Romagnani S. (2010). Evidence for a cross-talk between human neutrophils and Th17 cells. Blood.

[14] Burn G.L., Foti A., Marsman G., Patel D.F., Zychlinsky A. (2021). The Neutrophil. Immunity.

[15] Costa S., Bevilacqua D., Cassatella M.A., Scapini P. (2019). Recent advances on the crosstalk between neutrophils and B or T lymphocytes. Immunology.

[16] Puga I., Cols M., Barra C.M., He B., Cassis L., Gentile M., Comerma L., Chorny A., Shan M., Xu W. (2011). B cell–helper neutrophils stimulate the diversification and production of immunoglobulin in the marginal zone of the spleen. Nat. Immunol..

[17] Khoyratty T.E., Ai Z., Ballesteros I., Eames H.L., Mathie S., Martín-Salamanca S., Wang L., Hemmings A., Willemsen N., von Werz V. (2021). Distinct transcription factor networks control neutrophil-driven inflammation. Nat. Immunol..

[18] Montaldo E., Lusito E., Bianchessi V., Caronni N., Scala S., Basso-Ricci L., Cantaffa C., Masserdotti A., Barilaro M., Barresi S. (2022). Cellular and transcriptional dynamics of human neutrophils at steady state and upon stress. Nat. Immunol..

[19] Enthesitis: From Pathophysiology to Treatment—PubMed. https://pubmed.ncbi.nlm.nih.gov/29158573/.

[20] Ye P., Rodriguez F.H., Kanaly S., Stocking K.L., Schurr J., Schwarzenberger P., Oliver P., Huang W., Zhang P., Zhang J. (2001). Requirement of Interleukin 17 Receptor Signaling for Lung Cxc Chemokine and Granulocyte Colony-Stimulating Factor Expression, Neutrophil Recruitment, and Host Defense. J. Exp. Med..

[21] Yu J.J., Ruddy M.J., Wong G.C., Sfintescu C., Baker P.J., Smith J.B., Evans R.T., Gaffen S.L. (2007). An essential role for IL-17 in preventing pathogen-initiated bone destruction: Recruitment of neutrophils to inflamed bone requires IL-17 receptor–dependent signals. Blood.

[22] Tamassia N., Arruda-Silva F., Wright H.L., Moots R.J., Gardiman E., Bianchetto-Aguilera F., Gasperini S., Capone M., Maggi L., Annunziato F. (2019). Human neutrophils activated via TLR8 promote Th17 polarization through IL-23. J. Leukoc. Biol..

[23] Sakaguchi S., Takahashi T., Hata H., Nomura T., Sakaguchi N. (2003). SKG mice, a new genetic model of rheumatoid arthritis. Arthritis Res. Ther..

[24] Stavre Z., Bridgewood C., Zhou Q., Maeda Y., Huang T.-T., Karman J., Khan A., Giryes S., Sharif K., McGonagle D. (2022). A role for neutrophils in early enthesitis in spondyloarthritis. Arthritis Res. Ther..

[25] Crowe L.A.N., McLean M., Kitson S.M., Melchor E.G., Patommel K., Cao H.M., Reilly J.H., Leach W.J., Rooney B.P., Spencer S.J. (2019). S100A8 & S100A9: Alarmin mediated inflammation in tendinopathy. Sci. Rep..

[26] Schonthaler H.B., Guinea-Viniegra J., Wculek S.K., Ruppen I., Ximénez-Embún P., Guío-Carrión A., Navarro R., Hogg N., Ashman K., Wagner E.F. (2013). S100A8-S100A9 Protein Complex Mediates Psoriasis by Regulating the Expression of Complement Factor C3. Immunity.

[27] Turina M.C., Sieper J., Yeremenko N., Conrad K., Haibel H., Rudwaleit M., Baeten D., Poddubnyy D. (2014). Calprotectin serum level is an independent marker for radiographic spinal progression in axial spondyloarthritis. Ann. Rheum. Dis..

[28] Pruenster M., Kurz A.R.M., Chung K.-J., Cao-Ehlker X., Bieber S., Nussbaum C.F., Bierschenk S., Eggersmann T.K., Rohwedder I., Heinig K. (2015). Extracellular MRP8/14 is a regulator of β2 integrin-dependent neutrophil slow rolling and adhesion. Nat. Commun..

[29] De Wilde K., Martens A., Lambrecht S., Jacques P., Drennan M.B., Debusschere K., Govindarajan S., Coudenys J., Verheugen E., Windels F. (2017). A20 inhibition of STAT1 expression in myeloid cells: A novel endogenous regulatory mechanism preventing development of enthesitis. Ann. Rheum. Dis..

[30] McGonagle D., Marzo-Ortega H., O’Connor P., Gibbon W., Hawkey P., Henshaw K., Emery P. (2002). Histological assessment of the early enthesitis lesion in spondyloarthropathy. Ann. Rheum. Dis..

[31] Pachowsky M.L., Raimondo M.G., Xu C., Rauber S., Tascilar K., Labinsky H., Vogg M., Saad M.S.A., Simon D., Rech J. (2022). Concise report: A minimal-invasive method to retrieve and identify entheseal tissue from psoriatic arthritis patients. Ann. Rheum. Dis..

[32] Van Tubergen A., Weber U. (2012). Diagnosis and classification in spondyloarthritis: Identifying a chameleon. Nat. Rev. Rheumatol..

[33] Gong Y., Zheng N., Chen S.-B., Xiao Z.-Y., Wu M.-Y., Liu Y., Zeng Q.-Y. (2012). Ten years’ experience with needle biopsy in the early diagnosis of sacroiliitis. Arthritis Rheum..

[34] Appel H., Maier R., Wu P., Scheer R., Hempfing A., Kayser R., Thiel A., Radbruch A., Loddenkemper C., Sieper J. (2011). Analysis of IL-17+ cells in facet joints of patients with spondyloarthritis suggests that the innate immune pathway might be of greater relevance than the Th17-mediated adaptive immune response. Arthritis Res. Ther..

[35] Papagoras C., Tsiami S., Chrysanthopoulou A., Mitroulis I., Baraliakos X. (2022). Serum granulocyte-macrophage colony-stimulating factor (GM-CSF) is increased in patients with active radiographic axial spondyloarthritis and persists despite anti-TNF treatment. Arthritis Res. Ther..

[36] Regan-Komito D., Swann J.W., Demetriou P., Cohen E.S., Horwood N.J., Sansom S.N., Griseri T. (2020). GM-CSF drives dysregulated hematopoietic stem cell activity and pathogenic extramedullary myelopoiesis in experimental spondyloarthritis. Nat. Commun..

[37] Al-Osami M.H., Awadh N.I., Khalid K.B., Awadh A.I. (2020). Neutrophil/lymphocyte and platelet/lymphocyte ratios as potential markers of disease activity in patients with Ankylosing spondylitis: A case-control study. Hortic. Bras. Rheumatol..

[38] Karow F., Smiljanovic B., Grün J.R., Poddubnyy D., Proft F., Talpin A., Hue C., Boland A., Deleuze J.-F., Garchon H.-J. (2021). Monocyte transcriptomes from patients with axial spondyloarthritis reveal dysregulated monocytopoiesis and a distinct inflammatory imprint. Arthritis Res. Ther..

[39] Ruiz-Limon P., Ladehesa-Pineda M.L., Castro-Villegas M.D.C., Abalos-Aguilera M.D.C., Lopez-Medina C., Lopez-Pedrera C., Barbarroja N., Espejo-Peralbo D., González-Reyes J.A., Villalba J.M. (2020). Enhanced NETosis generation in radiographic axial spondyloarthritis: Utility as biomarker for disease activity and anti-TNF-α therapy effectiveness. J. Biomed. Sci..

[40] Papagoras C., Chrysanthopoulou A., Mitsios A., Ntinopoulou M., Tsironidou V., Batsali A.K., Papadaki H.A., Skendros P., Ritis K. (2021). IL-17A expressed on neutrophil extracellular traps promotes mesenchymal stem cell differentiation toward bone-forming cells in ankylosing spondylitis. Eur. J. Immunol..

[41] Mitsios A., Arampatzioglou A., Arelaki S., Mitroulis I., Ritis K. (2016). NETopathies? Unraveling the Dark Side of Old Diseases through Neutrophils. Front. Immunol..

[42] Jorch S.K., Kubes P. (2017). An emerging role for neutrophil extracellular traps in noninfectious disease. Nat. Med..

[43] Kendall M.J., Farr M., Meynell M.J., Hawkins C.F. (1973). Synovial fluid in ankylosing spondylitis. Ann. Rheum. Dis..

[44] Kruithof E., Baeten D., De Rycke L., Vandooren B., Foell D., Roth J., Cañete J.D., Boots A.M., Veys E.M., De Keyser F. (2005). Synovial histopathology of psoriatic arthritis, both oligo- and polyarticular, resembles spondyloarthropathy more than it does rheumatoid arthritis. Arthritis Res. Ther..

[45] Baeten D., Kruithof E., De Rycke L., Boots A.M., Mielants H., Veys E.M., De Keyser F. (2005). Infiltration of the synovial membrane with macrophage subsets and polymorphonuclear cells reflects global disease activity in spondyloarthropathy. Arthritis Res. Ther..

[46] Kruithof E., De Rycke L., Vandooren B., De Keyser F., FitzGerald O., McInnes I., Tak P.P., Bresnihan B., Veys E.M., Baeten D. (2006). Identification of synovial biomarkers of response to experimental treatment in early-phase clinical trials in spondylarthritis. Arthritis Rheum..

[47] Li B., Li G., Yang X., Song Z., Wang Y., Zhang Z. (2022). NETosis in Psoriatic Arthritis: Serum MPO–DNA Complex Level Correlates With Its Disease Activity. Front. Immunol..

[48] Lambert S., Hambro C.A., Johnston A., Stuart P.E., Tsoi L.C., Nair R.P., Elder J.T. (2019). Neutrophil Extracellular Traps Induce Human Th17 Cells: Effect of Psoriasis-Associated TRAF3IP2 Genotype. J. Investig. Dermatol..

[49] De Benedittis G., Latini A., Conigliaro P., Triggianese P., Bergamini A., Novelli L., Ciccacci C., Chimenti M.S., Borgiani P. (2022). A multilocus genetic study evidences the association of autoimmune-related genes with Psoriatic Arthritis in Italian patients. Immunobiology.

[50] Bierkarre H., Harder J., Cuthbert R., Emery P., Leuschner I., Mrowietz U., Hedderich J., McGonagle D., Gläser R. (2016). Differential expression of antimicrobial peptides in psoriasis and psoriatic arthritis as a novel contributory mechanism for skin and joint disease heterogeneity. Scand. J. Rheumatol..

[51] Frasca L., Palazzo R., Chimenti M.S., Alivernini S., Tolusso B., Bui L., Botti E., Giunta A., Bianchi L., Petricca L. (2018). Anti-LL37 Antibodies Are Present in Psoriatic Arthritis (PsA) Patients: New Biomarkers in PsA. Front. Immunol..

[52] Gracey E., Vereecke L., McGovern D., Fröhling M., Schett G., Danese S., De Vos M., Bosch F.V.D., Elewaut D. (2020). Revisiting the gut–joint axis: Links between gut inflammation and spondyloarthritis. Nat. Rev. Rheumatol..

[53] Fournier B.M., Parkos C.A. (2012). The role of neutrophils during intestinal inflammation. Mucosal Immunol..

[54] Mielants H., Veys E.M., Cuvelier C., De Vos M., Goemaere S., De Clercq L., Schatteman L., Gyselbrecht L., Elewaut D. (1995). The evolution of spondyloarthropathies in relation to gut histology. III. Relation between gut and joint. J. Rheumatol..

[55] Mielants H., Veys E.M., Cuvelier C., De Vos M., Goemaere S., De Clercq L., Schatteman L., Elewaut D. (1995). The evolution of spondyloarthropathies in relation to gut histology. II. Histological aspects. J. Rheumatol..

[56] Lin E., Lai H.-J., Cheng Y.-K., Leong K.-Q., Cheng L.-C., Chou Y.-C., Peng Y.-C., Hsu Y.-H., Chiang H.-S. (2020). Neutrophil Extracellular Traps Impair Intestinal Barrier Function during Experimental Colitis. Biomedicines.

[57] Spadoni I., Zagato E., Bertocchi A., Paolinelli R., Hot E., Di Sabatino A., Caprioli F., Bottiglieri L., Oldani A., Viale G. (2015). A gut-vascular barrier controls the systemic dissemination of bacteria. Science.

[58] Ciccia F., Guggino G., Rizzo A., Alessandro R., Luchetti M.M., Milling S., Saieva L., Cypers H., Stampone T., Di Benedetto P. (2017). Dysbiosis and zonulin upregulation alter gut epithelial and vascular barriers in patients with ankylosing spondylitis. Ann. Rheum. Dis..

[59] Rizzo A., Ferrante A., Guggino G., Ciccia F. (2017). Gut inflammation in spondyloarthritis. Best Pract. Res. Clin. Rheumatol..

[60] Tester A.M., Cox J.H., Connor A.R., Starr A.E., Dean R.A., Suarez-Puente X., López-Otín C., Overall C.M. (2007). LPS Responsiveness and Neutrophil Chemotaxis In Vivo Require PMN MMP-8 Activity. PLoS ONE.

[61] Van den Steen P.E., Proost P., Wuyts A., Van Damme J.O., Opdenakker G. (2000). Neutrophil gelatinase B potentiates interleukin-8 tenfold by aminoterminal processing, whereas it degrades CTAP-III, PF-4, and GRO-alpha and leaves RANTES and MCP-2 intact. Blood.

[62] Iking-Konert C., Ostendorf B., Sander O., Jost M., Wagner C., Joosten L., Schneider M., Hänsch G.M. (2005). Transdifferentiation of polymorphonuclear neutrophils to dendritic-like cells at the site of inflammation in rheumatoid arthritis: Evidence for activation by T cells. Ann. Rheum. Dis..

[63] Cypers H., Varkas G., Beeckman S., Debusschere K., Vogl T., Roth J., Drennan M., Lavric M., Foell D., Cuvelier C.A. (2016). Elevated calprotectin levels reveal bowel inflammation in spondyloarthritis. Ann. Rheum. Dis..

[64] Rizzo A., Guggino G., Ferrante A., Ciccia F. (2018). Role of Subclinical Gut Inflammation in the Pathogenesis of Spondyloarthritis. Front. Med..

[65] Schatteman L., Mielants H., Veys E.M., Cuvelier C., De Vos M., Gyselbrecht L., Elewaut D., Goemaere S. (1995). Gut inflammation in psoriatic arthritis: A prospective ileocolonoscopic study. J. Rheumatol..

[66] Macaluso F., Guggino G., Rizzo A., Ferrante A., Ciccia F. (2018). Histopathology of the gut in rheumatic diseases. Reumatismo.

[67] Mielants H., Veys E.M., De Vos M., Cuvelier C., Goemaere S., De Clercq L., Schatteman L., Elewaut D. (1995). The evolution of spondyloarthropathies in relation to gut histology. I. Clinical aspects. J. Rheumatol..

[68] Mantovani A., Cassatella M.A., Costantini C., Jaillon S. (2011). Neutrophils in the activation and regulation of innate and adaptive immunity. Nat. Rev. Immunol..

[69] Serhan C.N., Petasis N.A. (2011). Resolvins and Protectins in Inflammation Resolution. Chem. Rev..

[70] Kobayashi S.D., Voyich J.M., Whitney A.R., DeLeo F.R. (2005). Spontaneous neutrophil apoptosis and regulation of cell survival by granulocyte macrophage-colony stimulating factor. J. Leukoc. Biol..

[71] Arosa L., Camba-Gómez M., Conde-Aranda J. (2022). Neutrophils in Intestinal Inflammation: What We Know and What We Could Expect for the Near Future. Gastrointest. Disord..

[72] Brannigan A.E., O’connell R.P., Hurley H., O’neill A., Brady H.R., Fitzpatrick J.M., William R., Watson G. (2000). Neutrophil apoptosis is delayed in patients with inflammatory bowel disease. Shock.

[73] Ina K., Kusugami K., Hosokawa T., Imada A., Shimizu T., Yamaguchi T., Ohsuga M., Kyokane K., Sakai T., Nishio Y. (1999). Increased mucosal production of granulocyte colony-stimulating factor is related to a delay in neutrophil apoptosis in Inflammatory Bowel disease. J. Gastroenterol. Hepatol..

[74] Bassler K., Schulte-Schrepping J., Warnat-Herresthal S., Aschenbrenner A.C., Schultze J.L. (2019). The Myeloid Cell Compartment—Cell by Cell. Annu. Rev. Immunol..

[75] Qaiyum Z., Lim M., Inman R.D. (2021). The gut-joint axis in spondyloarthritis: Immunological, microbial, and clinical insights. Semin. Immunopathol..

[76] Griffiths C.E.M., Armstrong A.W., Gudjonsson J.E., Barker J.N.W.N. (2021). Psoriasis. Lancet.

[77] FitzGerald O., Ogdie A., Chandran V., Coates L.C., Kavanaugh A., Tillett W., Leung Y.Y., Dewit M., Scher J.U., Mease P.J. (2021). Psoriatic arthritis. Nat. Rev. Dis. Prim..

[78] Czerwińska J., Owczarczyk-Saczonek A. (2022). The Role of the Neutrophilic Network in the Pathogenesis of Psoriasis. Int. J. Mol. Sci..

[79] Naik H.B., Natarajan B., Stansky E., Ahlman M.A., Teague H., Salahuddin T., Ng Q., Joshi A.A., Krishnamoorthy P., Dave J. (2015). Severity of Psoriasis Associates With Aortic Vascular Inflammation Detected by FDG PET/CT and Neutrophil Activation in a Prospective Observational Study. Arter. Thromb. Vasc. Biol..

[80] Polat M., Bugdayci G., Kaya H., Oğuzman H. (2017). Evaluation of neutrophil-to-lymphocyte ratio and platelet-to-lymphocyte ratio in Turkish patients with chronic plaque psoriasis. Acta Dermatovenerol. Alp. Pannonica et Adriat..

[81] Teague H.L., Varghese N.J., Tsoi L.C., Dey A.K., Garshick M.S., Silverman J.I., Baumer Y., Harrington C.L., Stempinski E., Elnabawi Y.A. (2019). Neutrophil Subsets, Platelets, and Vascular Disease in Psoriasis. JACC Basic Transl. Sci..

[82] Toichi E., Tachibana T., Furukawa F. (2000). Rapid improvement of psoriasis vulgaris during drug-induced agranulocytosis. J. Am. Acad. Dermatol..

[83] Ikeda S., Takahashi H., Suga Y., Eto H., Etoh T., Okuma K., Takahashi K., Kanbara T., Seishima M., Morita A. (2013). Therapeutic depletion of myeloid lineage leukocytes in patients with generalized pustular psoriasis indicates a major role for neutrophils in the immunopathogenesis of psoriasis. J. Am. Acad. Dermatol..

[84] Yamanaka K., Umezawa Y., Yamagiwa A., Saeki H., Kondo M., Gabazza E., Nakagawa H., Mizutani H. (2014). Biologic therapy improves psoriasis by decreasing the activity of monocytes and neutrophils. J. Dermatol..

[85] Hwang T.-L. (2019). Neutrophils in Psoriasis. Front. Immunol..

[86] Bloomfield F.J., Young M.M. (1988). Enhanced chemiluminescence production by phagocytosing neutrophils in psoriasis. Inflammation.

[87] Dilek N., Dilek A.R., Taşkın Y., Erkinüresin T., Yalçın Ö., Saral Y. (2016). Contribution of myeloperoxidase and inducible nitric oxide synthase to pathogenesis of psoriasis. Postep. Dermatol. Allergol..

[88] Henry C.M., Sullivan G.P., Clancy D.M., Afonina I.S., Kulms D., Martin S.J. (2016). Neutrophil-Derived Proteases Escalate Inflammation through Activation of IL-36 Family Cytokines. Cell Rep..

[89] Xhindoli D., Pacor S., Benincasa M., Scocchi M., Gennaro R., Tossi A. (2016). The human cathelicidin LL-37—A pore-forming antibacterial peptide and host-cell modulator. Biochim. Biophys. Acta.

[90] Meyer-Hoffert U., Wingertszahn J., Wiedow O. (2004). Human Leukocyte Elastase Induces Keratinocyte Proliferation by Epidermal Growth Factor Receptor Activation. J. Investig. Dermatol..

[91] Loss-of-Function Myeloperoxidase Mutations Are Associated with Increased Neutrophil Counts and Pustular Skin Disease—PubMed. https://pubmed.ncbi.nlm.nih.gov/32758448/.

[92] Hau C.S., Kanda N., Tada Y., Shibata S., Uozaki H., Fukusato T., Sato S., Watanabe S. (2016). Lipocalin-2 exacerbates psoriasiform skin inflammation by augmenting T-helper 17 response. J. Dermatol..

[93] Schön M.P., Broekaert S.M.C., Erpenbeck L. (2017). Sexy again: The renaissance of neutrophils in psoriasis. Exp. Dermatol..

[94] Shao S., Fang H., Dang E., Xue K., Zhang J., Li B., Qiao H., Cao T., Zhuang Y., Shen S. (2019). Neutrophil Extracellular Traps Promote Inflammatory Responses in Psoriasis via Activating Epidermal TLR4/IL-36R Crosstalk. Front. Immunol..

[95] Lee K.H., Kronbichler A., Park D.D.-Y., Park Y., Moon H., Kim H., Choi J.H., Choi Y., Shim S., Lyu I.S. (2017). Neutrophil extracellular traps (NETs) in autoimmune diseases: A comprehensive review. Autoimmun. Rev..

[96] Hu S.C.-S., Yu H.-S., Yen F.-L., Lin C.-L., Chen G.-S., Lan C.-C.E. (2016). Neutrophil extracellular trap formation is increased in psoriasis and induces human β-defensin-2 production in epidermal keratinocytes. Sci. Rep..

[97] Wang W.-M., Jin H.-Z. (2020). Role of Neutrophils in Psoriasis. J. Immunol. Res..

[98] Ganguly D., Chamilos G., Lande R., Gregorio J., Meller S., Facchinetti V., Homey B., Barrat F.J., Zal T., Gilliet M. (2009). Self-RNA–antimicrobial peptide complexes activate human dendritic cells through TLR7 and TLR8. J. Exp. Med..

[99] Lande R., Gregorio J., Facchinetti V., Chatterjee B., Wang Y.-H., Homey B., Cao W., Wang Y.-H., Su B., Nestle F.O. (2007). Plasmacytoid dendritic cells sense self-DNA coupled with antimicrobial peptide. Nature.

[100] Lin A.M., Rubin C.J., Khandpur R., Wang J.Y., Riblett M., Yalavarthi S., Villanueva E.C., Shah P., Kaplan M.J., Bruce A.T. (2011). Mast Cells and Neutrophils Release IL-17 through Extracellular Trap Formation in Psoriasis. J. Immunol..

[101] Gao Y., Yao X., Zhai Y., Li L., Li H., Sun X., Yu P., Xue T., Li Y., Hu Y. (2021). Single cell transcriptional zonation of human psoriasis skin identifies an alternative immunoregulatory axis conducted by skin resident cells. Cell Death Dis..

[102] Rosenbaum J.T. (2015). Uveitis in spondyloarthritis including psoriatic arthritis, ankylosing spondylitis, and inflammatory bowel disease. Clin. Rheumatol..

[103] Rosenbaum J.T. (2019). The eye in spondyloarthritis✰. Semin. Arthritis Rheum..

[104] Khan M.A., Haroon M., Rosenbaum J.T. (2015). Acute Anterior Uveitis and Spondyloarthritis: More Than Meets the Eye. Curr. Rheumatol. Rep..

[105] Wakefield D., Yates W., Amjadi S., McCluskey P. (2016). HLA-B27 Anterior Uveitis: Immunology and Immunopathology. Ocul. Immunol. Inflamm..

[106] Chan C.-C., Li Q. (1998). Immunopathology of uveitis. Br. J. Ophthalmol..

[107] Zhao B., Chen W., Jiang R., Zhang R., Wang Y., Wang L., Gordon L., Chen L. (2015). Expression profile of IL-1 family cytokines in aqueous humor and sera of patients with HLA-B27 associated anterior uveitis and idiopathic anterior uveitis. Exp. Eye Res..

[108] Repo H., Leirisalo-Repo M., Koivuranta-Vaara P. (1984). Exaggerated inflammatory responsiveness plays a part in the pathogenesis of HLA-B27 linked diseases—Hypothesis. Ann. Clin. Res..

[109] Yamada M., Shichi H., Yuasa T., Tanouchi Y., Mimura Y. (1986). Superoxide in ocular inflammation: Human and experimental uveitis. J. Free. Radicals Biol. Med..

[110] Sedgwick J.B., Bergstresser P.R., Hurd E.R. (1980). Increased Granulocyte Adherence in Psoriasis and Psoriatic Arthritis. J. Investig. Dermatol..

[111] Iijima S., Iwata M., Otsuka F. (1996). Psoriatic Arthritis and Hypopyon-lridocyclitis. Possible mechanism of the association of psoriasis and anterior uveitis. Dermatology.

[112] Chang J.H., Hampartzoumian T., Everett B., Lloyd A., McCluskey P.J., Wakefield D. (2007). Changes in Toll-like Receptor (TLR)-2 and TLR4 Expression and Function but Not Polymorphisms Are Associated with Acute Anterior Uveitis. Investig. Opthalmology Vis. Sci..

[113] Erridge C., Pridmore A., Eley A., Stewart J., Poxton I.R. (2004). Lipopolysaccharides of *Bacteroides fragilis*, *Chlamydia trachomatis* and *Pseudomonas aeruginosa* signal via Toll-like receptor 2. J. Med Microbiol..

[114] Chang J.H., McCluskey P., Wakefield D. (2006). Toll-like receptors in ocular immunity and the immunopathogenesis of inflammatory eye disease. Br. J. Ophthalmol..

[115] Kawakami T., Ohashi S., Kawa Y., Takahama H., Ito M., Soma Y., Mizoguchi M. (2004). Elevated Serum Granulocyte Colony-Stimulating Factor Levels in Patients With Active Phase of Sweet Syndrome and Patients With Active Behçet Disease: Implication in neutrophil apoptosis dysfunction. Arch. Dermatol..

[116] The Antiapoptotic Protein Mcl-1 Is Essential for the Survival of Neutrophils but Not Macrophages—PubMed. https://pubmed.ncbi.nlm.nih.gov/17062731/.

[117] Increased Production of Pro-Inflammatory Cytokines and Enhanced T Cell Responses after Activation of Human Dendritic Cells with IL-1 and CD40 Ligand—PubMed. https://pubmed.ncbi.nlm.nih.gov/12385649/.

[118] Goldberg G.L., Cornish A.L., Murphy J., Pang E.S., Lim L.L., Campbell I.K., Scalzo-Inguanti K., Chen X., McMenamin P.G., Maraskovsky E. (2016). G-CSF and Neutrophils Are Nonredundant Mediators of Murine Experimental Autoimmune Uveoretinitis. Am. J. Pathol..

[119] Iijima S., Iwata M., Otsuka F. (1996). Rheological analysis of peripheral blood neutrophils in a patient with cutaneous and arthropathic psoriasis accompanying hypopyon-iridocyclitis. Australas. J. Dermatol..

[120] Morton A.M., Sefik E., Upadhyay R., Weissleder R., Benoist C., Mathis D. (2014). Endoscopic photoconversion reveals unexpectedly broad leukocyte trafficking to and from the gut. Proc. Natl. Acad. Sci. USA.

[121] Hysa E., Cutolo C.A., Gotelli E., Pacini G., Schenone C., Kreps E.O., Smith V., Cutolo M. (2021). Immunopathophysiology and clinical impact of uveitis in inflammatory rheumatic diseases: An update. Eur. J. Clin. Investig..

[122] Németh T., Sperandio M., Mócsai A. (2020). Neutrophils as emerging therapeutic targets. Nat. Rev. Drug Discov..

[123] De Benedetti F., Ruperto N., Baildam E., Burgos-Vargas R., Horneff G., Huppertz H.I., Minden K., Myones B.L., Onel K., Wang J. (2014). A14: Neutropenia With Tocilizumab Treatment Is Not Associated With Increased Infection Risk in Patients with Systemic Juvenile Idiopathic Arthritis. Arthritis Rheumatol..

[124] Shovman O., Shoenfeld Y., Langevitz P. (2015). Tocilizumab-induced neutropenia in rheumatoid arthritis patients with previous history of neutropenia: Case series and review of literature. Immunol. Res..

[125] Strand V., Dikranian A., Beal J., Kwok K., Krishnaswami S., Wood S., Nduaka C. (2014). Analysis of early neutropenia, clinical response, and serious infection events in patients receiving tofacitinib for rheumatoid arthritis. Arthritis & Rheumatology.

[126] Wigerblad G., Cao Q., Brooks S., Naz F., Gadkari M., Jiang K., Gupta S., O’Neil L., Dell’Orso S., Kaplan M.J. (2022). Single-Cell Analysis Reveals the Range of Transcriptional States of Circulating Human Neutrophils. J. Immunol..

[127] Burmester G.R., McInnes I.B., Kremer J., Miranda P., Korkosz M., Vencovsky J., Rubbert-Roth A., Mysler E., Sleeman M.A., Godwood A. (2017). A randomised phase IIb study of mavrilimumab, a novel GM–CSF receptor alpha monoclonal antibody, in the treatment of rheumatoid arthritis. Ann. Rheum. Dis..

[128] Cid M.C., Unizony S.H., Blockmans D., Brouwer E., Dagna L., Dasgupta B., Hellmich B., Molloy E., Salvarani C., Trapnell B.C. (2022). Efficacy and safety of mavrilimumab in giant cell arteritis: A phase 2, randomised, double-blind, placebo-controlled trial. Ann. Rheum. Dis..

[129] Weinblatt M.E., McInnes I.B., Kremer J.M., Miranda P., Vencovsky J., Guo X., White W.I., Ryan P.C., Godwood A., Albulescu M. (2018). A Randomized Phase II b Study of Mavrilimumab and Golimumab in Rheumatoid Arthritis. Arthritis Rheumatol..

[130] Buckley C.D., Simón-Campos J.A., Zhdan V., Becker B., Davy K., Fisheleva E., Gupta A., Hawkes C., Inman D., Layton M. (2020). Efficacy, patient-reported outcomes, and safety of the anti-granulocyte macrophage colony-stimulating factor antibody otilimab (GSK3196165) in patients with rheumatoid arthritis: A randomised, phase 2b, dose-ranging study. Lancet Rheumatol..

[131] Taylor P.C., Saurigny D., Vencovsky J., Takeuchi T., Nakamura T., Matsievskaia G., Hunt B., Wagner T., Souberbielle B., for the NEXUS Study Group (2019). Efficacy and safety of namilumab, a human monoclonal antibody against granulocyte-macrophage colony-stimulating factor (GM-CSF) ligand in patients with rheumatoid arthritis (RA) with either an inadequate response to background methotrexate therapy or an inadequate response or intolerance to an anti-TNF (tumour necrosis factor) biologic therapy: A randomized, controlled trial. Arthritis Res. Ther..

[132] Papp K., Gooderham M., Jenkins R., Vender R., Szepietowski J., Wagner T., Hunt B., Souberbielle B., on behalf of the NEPTUNE investigators, The NEPTUNE investigators (2019). Granulocyte–macrophage colony-stimulating factor (GM-CSF) as a therapeutic target in psoriasis: Randomized, controlled investigation using namilumab, a specific human anti-GM-CSF monoclonal antibody. Br. J. Dermatol..

[133] Gaffen S.L., Jain R., Garg A.V., Cua D.J. (2014). The IL-23–IL-17 immune axis: From mechanisms to therapeutic testing. Nat. Rev. Immunol..

[134] Martínez A.V.C., Chiva L.M., Villarino M.R. (2021). Granulocyte and monocyte/macrophage apheresis for the treatment of immune-mediated inflammatory arthropathies: Case reports. Drugs Context.

[135] Chen X.-L., Mao J.-W., Wang Y.-D. (2020). Selective granulocyte and monocyte apheresis in inflammatory bowel disease: Its past, present and future. World J. Gastrointest. Pathophysiol..

[136] Gubatan J., Keyashian K., Rubin S.J., Wang J., Buckman C.A., Sinha S. (2021). Anti-Integrins for the Treatment of Inflammatory Bowel Disease: Current Evidence and Perspectives. Clin. Exp. Gastroenterol..

[137] Schön M.P., Zollner T.M., Boehncke W.-H. (2003). The Molecular Basis of Lymphocyte Recruitment to the Skin: Clues for Pathogenesis and Selective Therapies of Inflammatory Disorders. J. Investig. Dermatol..

[138] Jayne D.R., Merkel P.A., Schall T.J., Bekker P. (2021). Avacopan for the Treatment of ANCA-Associated Vasculitis. N. Engl. J. Med..

[139] Huizenga N., Zonozi R., Rosenthal J., Laliberte K., Niles J.L., Cortazar F.B. (2020). Treatment of Aggressive Antineutrophil Cytoplasmic Antibody–Associated Vasculitis With Eculizumab. Kidney Int. Rep..

[140] Shawky A.M., Almalki F.A., Abdalla A.N., Abdelazeem A.H., Gouda A.M. (2022). A Comprehensive Overview of Globally Approved JAK Inhibitors. Pharmaceutics.

[141] Tanaka Y., Millson D., Iwata S., Nakayamada S. (2021). Safety and efficacy of fostamatinib in rheumatoid arthritis patients with an inadequate response to methotrexate in phase II OSKIRA-ASIA-1 and OSKIRA-ASIA-1X study. Rheumatology.

[142] Walker A., Erwig L., Foster K., Nevin K., Wenzel J., Worm M., Williams N., Ratia N., Hoang B., Schneider-Merck T. (2021). Safety, pharmacokinetics and pharmacodynamics of a topical SYK inhibitor in cutaneous lupus erythematosus: A double-blind Phase Ib study. Exp. Dermatol..

[143] Keating G.M. (2017). Apremilast: A Review in Psoriasis and Psoriatic Arthritis. Drugs.

[144] Taylor P.C., van der Heijde D., Landewé R., McCue S., Cheng S., Boonen A. (2021). A Phase III Randomized Study of Apremilast, an Oral Phosphodiesterase 4 Inhibitor, for Active Ankylosing Spondylitis. J. Rheumatol..

[145] Gerriets V., Goyal A., Khaddour K. (2022). Tumor Necrosis Factor Inhibitors. StatPearls.

[146] Choy E.H., De Benedetti F., Takeuchi T., Hashizume M., John M.R., Kishimoto T. (2020). Translating IL-6 biology into effective treatments. Nat. Rev. Rheumatol..

[147] Sandborn W.J., Bhandari B.R., Randall C., Younes Z.H., Romanczyk T., Xin Y., Wendt E., Chai H., McKevitt M., Zhao S. (2018). Andecaliximab [Anti-matrix Metalloproteinase-9] Induction Therapy for Ulcerative Colitis: A Randomised, Double-Blind, Placebo-Controlled, Phase 2/3 Study in Patients With Moderate to Severe Disease. J. Crohns Colitis.

[148] Schreiber S., Siegel C.A., Friedenberg K.A., Younes Z.H., Seidler U., Bhandari B.R., Wang K., Wendt E., McKevitt M., Zhao S. (2018). A Phase 2, Randomized, Placebo-Controlled Study Evaluating Matrix Metalloproteinase-9 Inhibitor, Andecaliximab, in Patients With Moderately to Severely Active Crohn’s Disease. J. Crohn’s Colitis.

[149] Goldberg E.L., Asher J.L., Molony R.D., Shaw A.C., Zeiss C.J., Wang C., Morozova-Roche L.A., Herzog R.I., Iwasaki A., Dixit V.D. (2017). β-Hydroxybutyrate Deactivates Neutrophil NLRP3 Inflammasome to Relieve Gout Flares. Cell Rep..

[150] Stefania S., Colia R., Cinzia R., Corrado A., Cantatore F.P. (2021). Off-label use of anti-IL-1 drugs in rheumatic diseases. Int. J. Immunopathol. Pharmacol..

[151] Nakamura A., Zeng F., Nakamura S., Reid K.T., Gracey E., Lim M., Leng L., Jo S., Park Y.-S., Kusuda M. (2021). Macrophage migration inhibitory factor drives pathology in a mouse model of spondyloarthritis and is associated with human disease. Sci. Transl. Med..

[152] Peiseler M., Kubes P. (2019). More friend than foe: The emerging role of neutrophils in tissue repair. J. Clin. Investig..

[153] The role of IL-17A in axial spondyloarthritis and psoriatic arthritis: Recent advances and controversies—PubMed. https://pubmed.ncbi.nlm.nih.gov/31278139/.

[154] Tamassia N., e Silva F.A., Calzetti F., Lonardi S., Gasperini S., Gardiman E., Bianchetto-Aguilera F., Gatta L.B., Girolomoni G., Mantovani A. (2018). A Reappraisal on the Potential Ability of Human Neutrophils to Express and Produce IL-17 Family Members In Vitro: Failure to Reproducibly Detect It. Front. Immunol..

